# Correction to “Enrichment of 1.0 Nanometer Diameter Single-Chirality Single-Walled Carbon Nanotubes Dictated by Conjugated Polymer Characteristics: Implications for High-Performance Thin-Film Transistors”

**DOI:** 10.1021/acsanm.5c02805

**Published:** 2025-08-01

**Authors:** Jianying Ouyang, Homin Shin, Paul Finnie, Jianfu Ding, Zhao Li, Brendan Mirka, Patrick R. L. Malenfant

In Figure [Fig figS2] of the Supporting Information of the original paper, the alignment of subfigures was previously distorted and now has been corrected.

**S2 figS2:**
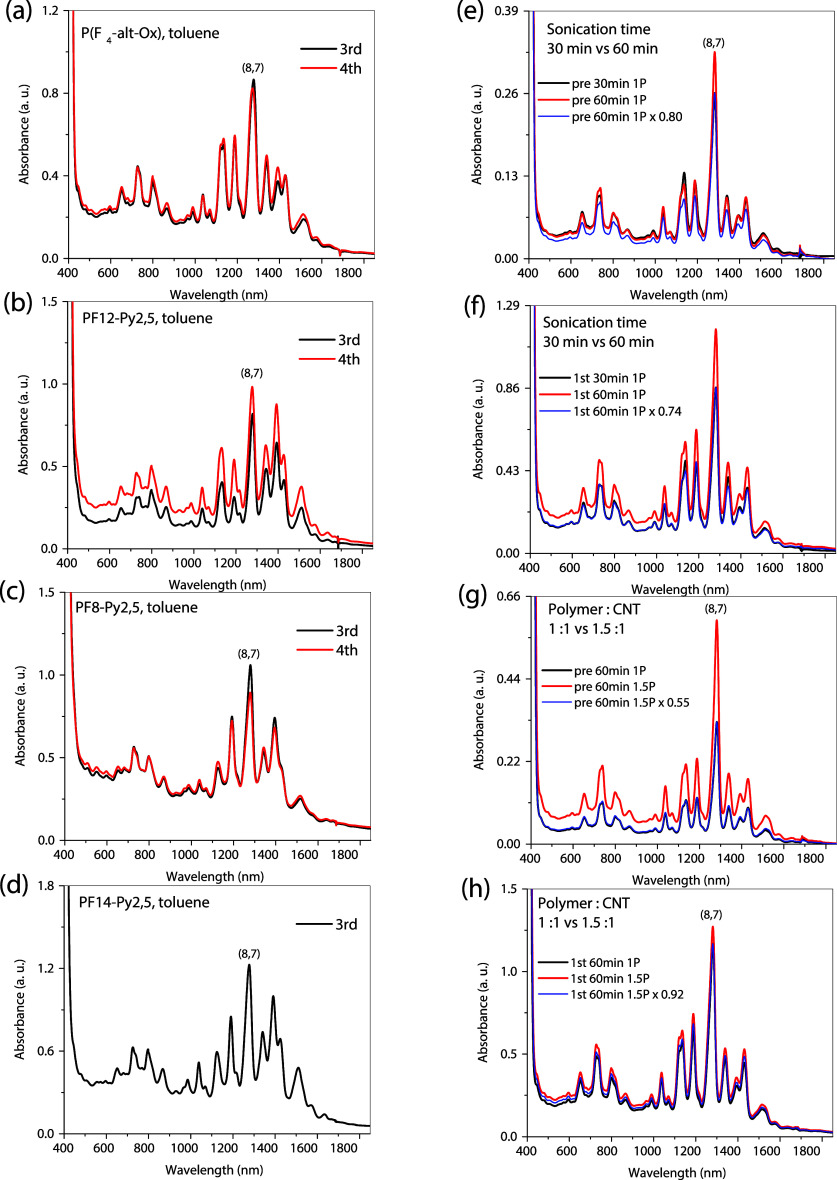
(left) Absorption spectra of enriched products after initial 3 cycles by various fluorene copolymers in toluene. (a) P­(F_4_-*alt*-Ox); (b) PF12-Py2,5; (c) PF8-Py2,5; and (d) PF14-Py2,5. (right) Control experiments using P­(F_4_-*alt*-Ox) with a sonication time of 60 min vs regular 30 min in pre-cycle (e) and 1st-cycle (f), and a polymer concentration of 1.0 mg/mL (1.5P:1CNT) vs regular 0.67 mg/mL (1P:1CNT) in pre-cycle (g) and 1st-cycle (h).

